# Potential Therapeutic Targets of Formononetin, a Type of Methoxylated Isoflavone, and Its Role in Cancer Therapy through the Modulation of Signal Transduction Pathways

**DOI:** 10.3390/ijms24119719

**Published:** 2023-06-03

**Authors:** Saleh A. Almatroodi, Ahmad Almatroudi, Amjad Ali Khan, Arshad Husain Rahmani

**Affiliations:** 1 Department of Medical Laboratories, College of Applied Medical Sciences, Qassim University, Buraydah 51542, Saudi Arabia; 2 Department of Basic Health Sciences, College of Applied Medical Sciences, Qassim University, Buraydah 51542, Saudi Arabia

**Keywords:** formononetin, apoptosis, inflammation, signal transduction, cancer therapy

## Abstract

Cancer is one of the main causes of death in all developed and developing countries. Various factors are involved in cancer development and progression, including inflammation and alterations in cellular processes and signaling transduction pathways. Natural compounds have shown health-promoting effects through their antioxidant and anti-inflammatory potential, having an important role in the inhibition of cancer growth. In this regard, formononetin, a type of isoflavone, plays a significant role in disease management through the modulation of inflammation, angiogenesis, cell cycle, and apoptosis. Furthermore, its role in cancer management has been proven through the regulation of different signal transduction pathways, such as the signal transducer and activator of transcription 3 (STAT 3), Phosphatidyl inositol 3 kinase/protein kinase B (PI3K/Akt), and mitogen activating protein kinase (MAPK) signaling pathways. The anticancer potential of formononetin has been reported against various cancer types, such as breast, cervical, head and neck, colon, and ovarian cancers. This review focuses on the role of formononetin in different cancer types through the modulation of various cell signaling pathways. Moreover, synergistic effect with anticancer drugs and methods to improve bioavailability are explained. Thus, detailed studies based on clinical trials are required to explore the potential role of formononetin in cancer prevention and treatment.

## 1. Introduction

Cancer is one of the major causes of death globally, and its incidence is increasing continuously. According to cancer statistics in 2022, 1,918,030 new cancer cases and 609,360 cancer-associated deaths are anticipated [1]. The therapeutic selections against cancer consist of radiation therapy, surgical procedures, chemotherapy, as well as specific-tissue-targeted therapy [2]. The current mode of treatment module causes adverse complications and the cost of treatment is also high. However, the search of effective, safe, and less expensive treatment modules is of prime interest for health science researchers. In this regard, natural compounds and their bioactive constituents have proven health-promoting effects and also play role in the prevention of diseases, including cancer.

**Figure 1:**
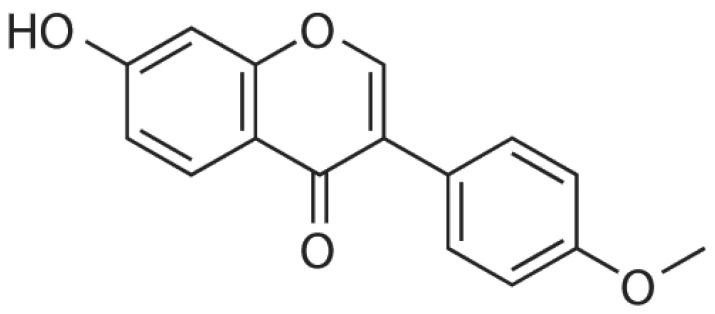
Chemical structure of formononetin.

Formononetin, a methoxylated isoflavone (7-hydroxy-3-(4-methoxypheny)-4H-1-benzopyran-4-one) (Figure 1), is a chief ingredient isolated from red clover as well as the Chinese plant *Astragalus membranaceus* [3], and it belongs to the isoflavonoid group of phytoestrogens [4,5].

The role of formononetin has been described in different cancers through the regulation of different cellular processes and molecular signaling pathways. Recently, the role of formononetin has been reviewed in different types of cancers and specific molecular targets [6,7]. This article explains the anti-tumor potential of formononetin on numerous cancers and its mechanism of action in cancer prevention by means of modulating numerous cell-signaling molecules.

## 2. Mechanism of Action of Formononetin

Formononetin controls the development and progression of cancer through different mechanisms, such as the inhibition of angiogenesis, induction of apoptosis, cell cycle arrest, inhibition of inflammation, and activation of tumor suppressor genes. Furthermore, formononetin plays a significant role in different cell-signaling pathways, such as P38 MAPK, PI3K/Akt, STAT3, and EFGR. 

### 2.1. Effect of Formononetin on Cellular Processes

#### 2.1.1. Cell Cycle

**Figure 2:**
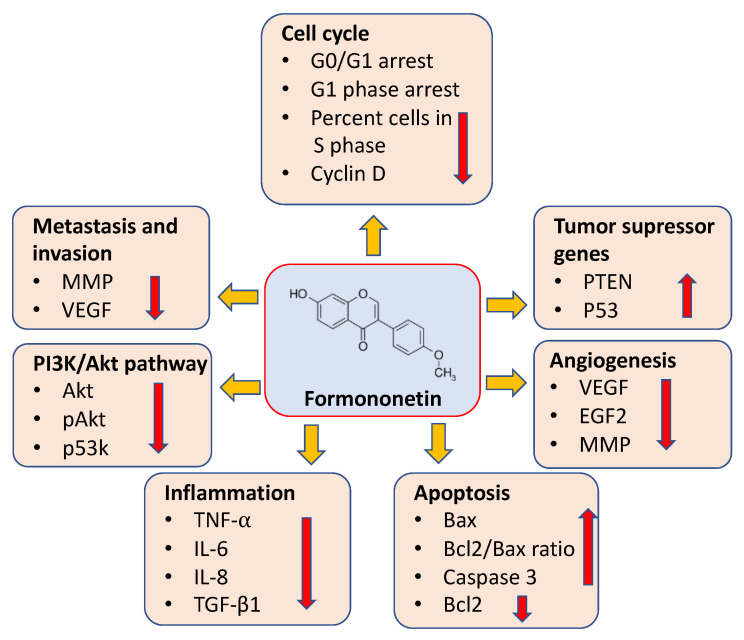
Mechanism of action of formononetin through the modulation of cell signaling pathways, such as cell cycle arrest, the activation of tumor suppressor genes, inhibition of angiogenesis, induction of apoptosis, inhibition of inflammation, and invasion.

The alteration of the cell cycle plays a major role in cancer development as well as progression. Thus, the normal functioning of the cell cycle as well as the induction of cell cycle arrest are valuable approaches to managing cancer (Figure 2). In this regard, formononetin has been shown to have a significant contribution in cancer inhibition through cell cycle arrest by affecting different cell cycle mediators, such as cyclin D1 and cyclin DK4 (CDK4) [8]. It was observed that formononetin-treated cells had a slow increase in the percentage of G0/G1 phase, demonstrating that formononetin showed inhibitory potential in prostate cancer cell growth through the initiation of cell cycle arrest [8] (Table 1).

The effect of formononetin on the cell cycle was investigated and it was noticed that cells that were treated with formononetin accumulated at the G0/G1 phase; the proportion of cells experiencing the G0/G1 phase increased to 21.6% after the cells were treated with formononetin (80 μM). The results propose that formononetin efficiently causes the initiation of cell cycle arrest [9].

Treatment of A549 cells with formononetin enhanced the proportions of cells in the G1-phase, as well reduced the number of cells in the S phase [10].

A recent study reported that cancer cells (SW1116 and HCT116) treated with various formononetin concentrations caused an increase in the amount in the G0/G1 phase; the proportion of cells experiencing this phase increased to 79.7% following subsequent treatment with a higher concentration of formononetin (100 µM) [11].

#### 2.1.2. Apoptosis

Formononetin has a significant effect on the mediators of apoptosis, such as miR-375, and RASD1 [12,13]. Formononetin-treated tumor-bearing animals showed reduced tumor mass and intercellular miR-375 expression. In the meantime, immuno-labeled cells and proteins of caspase-3, Bax, and Apaf-1 in formononetin-treated animals were enhanced dose-dependently [12]. 

The levels of Bax and RASD1 protein in prostate cancer cells (DU-145) were enhanced after treatment with several concentrations of formononetin. However, the levels of the Bcl-2 protein reduced in a dose-dependent fashion [13] (Table 1). Formononetin treatment showed a role in decreasing Bcl-2 protein and increasing Bax protein expressions in prostatic adenocarcinomas [14]. 

Formononetin (high concentrations) suggestively caused the induction of cell apoptosis and inhibited the proliferation of these cancer cells [15] (Table 1).

Osteosarcoma cells were treated with several doses of formononetin. The percentage of apoptotic cells was assessed and it was noticed that the number of early apoptotic cells slowly increased [16], and other study based on prostate cancer showed the role of formononetin in the induction of apoptosis [17]. Moreover, formononetin reduced the expression levels of pIGF-1 R protein as well as the increased Bax mRNA and protein expression levels in a dose-dependent way [18].

#### 2.1.3. Angiogenesis

Angiogenesis shows a noteworthy role in cancer development and progression. In the tumor matrix, matrix metalloproteinases (MMPs) play a vital role in the release of angiogenic factors, such as vascular endothelial growth factor (VEGF). Formononetin plays a significant role in the inhibition of angiogenesis by targeting different angiogenic factors, such as VEGF, basic fibroblast growth factor (bFGF), and MMPs. 

In formononetin-treated HCT-116 cells, both the gene and protein expressions of VEGF were downregulated, demonstrating the suppression of angiogenesis. The results show that formononetin significantly decreases the serum VEGF levels by nearly 50% as compared to the control group. The excised tissues were examined for the expression of VEGF, which exhibited a decreased expression of VEGF in formononetin-treated group tissues as compared to the control group [19] (Table 1). Xenograft models of breast cancer showed that formononetin possesses the growth-inhibitory potential associated with tumor angiogenesis inhibition. Likewise, formononetin increased the effect of the VEGFR2 inhibitor, sunitinib, on the inhibition of tumor growth [20].

#### 2.1.4. Inflammation

An association between the development of cancer and inflammation has long-been understood [21]. Chronic inflammation increases the risk of developing cancer in several ways, including tissue remodeling, structural damage to tissues and proteins, and DNA modifications caused by oxidative stress [22,23]. Macrophages are chiefly found near the chronic inflammatory microenvironment. These macrophages with other leukocytes produce a high level of reactive nitrogen species (RNS) and reactive oxygen species (ROS) to fight the infection [24]. During continuous tissue damage and cellular proliferation, the persistence of infection-fighting agents is injurious. It leads to the production of mutagenic agents, such as peroxynitrite. It binds with DNA and leads to mutations in stroma and epithelial cells. T-lymphocytes and macrophages release the TNF-α macrophage migration-inhibitory factor to worsen DNA damage [25]. Uncontrolled DNA damage is a major factor in the development of neoplasms. 

Around 25% of human cancers are associated to chronic inflammation, as well as bacterial/viral infections [26]. Formononetin specifically suppresses cytokine-induced apoptotic signaling and INS-1 cell death, which are both caused by IL-1 [27]. It was reported that formononetin exposure markedly decreased the expressions of IL-6, IL-8, HIF-1α, TNF-α, and TGF-β1, in hypoxic conditions. Formononetin anti-inflammatory properties have also been shown to have the potential to reduce tumor volumes when administered intragastrically [28] (Table 1).

### 2.2. Effect of Formononetin on Cell Signaling Pathways

#### 2.2.1. P38 MAPK Signaling Pathway

The mitogen-activated protein kinase (MAPK) pathway is an intracellular signal transduction pathway that regulates a plethora of cellular processes, such as cell growth, cell differentiation, cell proliferation, migration, as well as apoptosis, in response to numerous extracellular stimuli [29,30,31]. The activation of p38 MAPKs has been described to contribute to the epithelial mesenchymal transition of cells in the primary tumor, to the acquisition of invasion as well as migrating abilities, and to the extravasation of migrating tumor cells [32,33]. 

The phosphorylated level of p38 in prostate cancer cells was activated via the formononetin treatment, while endogenous Akt phosphorylation was blocked. Furthermore, the result shows that formononetin has an influence on prostate cancer, in which the underlying mechanisms are related with controlling the p38/Akt pathway [14].

The mechanism of action of formononetin in the nasopharyngeal carcinoma cells was investigated. It was noticed that this compound meaningfully decreased Akt phosphorylation, whereas it enhanced the phosphorylation of c-Jun N-terminal kinase/stress-activated protein kinase (JNK/SAPK) as well as p38MAPK [34] (Table 1). 

#### 2.2.2. PI3K/Akt Signaling Pathway

The endogenous Akt phosphorylation was blocked by formononetin treatment, as this compound showed an anticarcinogenic potential on prostate cancer [14]. Breast cancer-based results reported that immunoblotting and immunofluorescence assays showed that formononetin was very effective in suppressing the phosphorylation of Akt and PI3K [35] (Table 1).

A colon cancer-based study reported that formononetin inhibited cell proliferation and invasion via the inhibition of cyclin D1 and MMP-2/9 expression through p-STAT3, p-PI3K, and p-Akt inactivation [11]. Formononetin showed an effective role in cell cycle arrest at the G0/G1 phase by inactivating IGF1/IGF1R-PI3K/Akt pathways and diminishing cyclin D1 mRNA and its protein expression [9].

#### 2.2.3. Tumor Suppressor Gene

The phosphatase and tensin homolog (*PTEN*) is a vital tumor suppressor gene that inhibits cell growth and increases cellular sensitivity to apoptosis [36]. The altered expression of the PTEN protein has been found in many tumors [37,38].

A bladder cancer-based study reported that miR-21 expression was significantly reduced in formononetin-exposed bladder cancer cells, followed by an increase in PTEN, as well as p-Akt downregulation [39] (Table 1). Another study based on osteosarcoma reported that qRT-PCR analysis on miR-214-3p as well as PTEN expressions after treatment with formononetin was examined, and the results show the miR-214-3p level was noticeably reduced and PTEN level was evidently improved by formononetin treatment [40] (Table 1).

Some results show that the total p53 expression showed an increasing trend in response to formononetin treatment. Additionally, the phosphorylation levels of p53 at Ser20 and Ser15 were meaningfully upregulated in a dose-dependent way following treatment with formononetin at various concentrations. Furthermore, the transactivation ability of p53 was significantly elevated following the treatment of formononetin. These results show that p53 participates in formononetin effects on apoptotic induction in non-small cell lung cancer cells [10].

#### 2.2.4. Signal Transducer and Activator of Transcription 3 (STAT3)

The JAK/STAT pathway components are receptor tyrosine kinase (RTK), JAK, and STAT proteins [41,42]. In addition to these main components, some other effector proteins have been recognized to contribute to at least a subset of JAK/STAT signaling actions, such as interferon (IFN) regulatory factor 9 (IRF-9) in the case of type-I IFN-activated JAK/STAT signaling and signal-transducing adapter molecules (STAMs), which are adapter molecules with retained VHS and SH3 domains [43].

Formononetin, a natural product isolated from *A. membranaceus*, suppresses PD-L1 protein synthesis through a decrease in STAT3 and MYC protein expressions. Additionally, formononetin evidently decreases the expression of the MYC protein through the RAS/ERK signaling pathway and inhibits STAT3 activation via the JAK1/STAT3 pathway [44].

Remarkably, formononetin suppressed constitutive STAT3 and STAT5 activations. Altogether, the findings show that formononetin exhibits noteworthy anticancer potential in multiple myelomas that may be chiefly mediated via the ROS-regulated inhibition of STAT3 as well as the STAT5 signaling cascade [45] (Table 1). A colon cancer-based study reported that the inhibitory effects of formononetin were related with STAT3 and phosphatidylinositol-3-kinase (PI3K)/protein kinase B (AKT) signaling pathways [11] (Table 1).

**Table 1:** Mechanism of action of formononetin in cancer management through the modulation of cellular processes.

Cellular Processes/ Molecular Signaling Pathways	Cancer	Cell Lines	Mechanism/Outcome of the Study	Refs.
Cell cycle	Prostate cancer	PC-3 and DU-145	It prevented cancer cell proliferation by initiating cell cycle capture at the G0/G1 phase	[8]
	Breast cancer	MCF-7	It had a role in cell cycle arrest at the G0/G1 phase	[9]
	Lung cancer	A549 and NCI-H23	This compound induced G1-phase cell cycle arrest	[10]
	Colon cancer	SW1116 and HCT116	The proportion of cells in the G0–G1 stage increased with formononetin treatment (20, 50, and 100 µM concentration)	[11]
Apoptosis	Prostate cancer	DU-145	Formononetin inhibited the levels of Bcl-2 protein and caused the induction of the activation of RASD1 as well as Bax in a dose-dependent way	[13]
		PC-3	The treatment contributed to an elevated Bax expression and reduced Bcl-2 protein level	[14]
	Bone cancer	U2SO	Higher concentrations significantly reduced Bcl-2 expression in comparison to the control group	[15]
		U2OS	It inhibited the growth of human osteosarcoma cells by inducing apoptosis	[16]
	Prostate cancer	LNCaP and PC-3	The ERK1/2 MAPK signaling pathway was inactivated, which elevated the expression of Bcl-2-associated X (Bax) mRNA	[17]
		PC-3	This compound decreased the expression levels of the pIGF-1 R protein as well as increasing Bax mRNA	[18]
Angiogenesis	Colon cancer	HCT-116	Colon cancer cells treated with formononetin showed a decreased expression of the VEGF gene and protein	[19]
Inflammation	Multiple myeloma	U266	TNF-α, TGF-β1, IL-6, and IL-8 were upregulated in response to hypoxia, but formononetin prevented this	[28]
p38/Akt	Prostate cancer	PC-3	This compound showed an anticarcinogenic effect, with potential mechanisms leading to the elevation of the Bax/Bcl-2 ratio	[14]
MAPK	Nasopharyngeal cancer	CNE1	The mitochondrial apoptotic pathway may be regulated by the PI3K/Akt and MAPK cascades, which would facilitate the anticancer impacts of formononetin	[34]
PI3K/Akt	Breast cancer	MDA-MB-231 and 4T1	By reducing the expression of MMP-2,9 via the PI3K/AKT signaling pathway, formononetin reduced both the migration and invasion of cancer cells	[35]
	Colon cancer	SW1116, and HCT116	The treatment significantly decreased p-PI3K and p-AKT protein expressions	[11]
	Breast cancer	MCF-7	By deactivating the IGF1/IGF1R-PI3K/Akt pathways, formononetin demonstrated a role in cell cycle arrest	[9]
PTEN	Bladder cancer	T24	miR-21 expression was significantly reduced and this was followed by an increase in PTEN and a decrease in p-Akt	[39]
	Bone cancer	MG-63	Formononetin increased PTEN expression, reduced miR-214-3p levels, and decreased cell viability while promoting apoptosis	[40]
p53	Lung cancer	A549	Expression level of p53 was upregulated when cells wereexposed to formononetin	[10]
Signal transducer and activator of transcription 3 (STAT3)	Multiple myeloma	U266	Both constitutive p-STAT3 (Tyr705) and p-STAT3 (Ser727) levels were substantially reduced upon FT treatment	[45]
	Colon cancer	W1116 and HCT116	Suppressive potential of formononetin on colon carcinoma cell proliferation and invasion was noticed, including the inhibition of PI3K/AKT as well as STAT3 signaling pathways	[11]

#### 2.2.5. MMPs Role in Cancer Development

A vital step in invasion is the disassembly of the extra cellular matrix and its constituents via enzymes, such as MMPs [46]. MMPs play a significant role in cell survival, proliferation, immune response, angiogenesis, and invasion [47,48]. As several MMPs are expressed in all tumors, it is evident at present that each MMP can contribute to distinct vascular events in the same tumor [49].

Malignant cells possess key hallmarks as uncontrolled growth potentials and the capability to invade surrounding tissues [50]. However, invasion and metastasis inhibition are a critical step towards cancer prevention and treatment. Formononetin inhibited metastasis as well as cell invasion via the modulation of various cell signaling pathways. It was reported that formononetin meaningfully inhibited cell invasiveness in a concentration-dependent manner. Overall, these finding show the strong anti-proliferation and anti-invasion effects of formononetin on colon cancer cells [11]. Formononetin decreased the expression of MMP-2, -9 and boosted the expression of tissue inhibitors of MMP-1 and -2 [35]. Formononetin decreased the expression of the important pro-angiogenic factors containing MMPs. In the formononetin-treated group, it was observed that the number of proliferating cells and tumor size were significantly reduced in the tumor tissues [19].

#### 2.2.6. Epidermal Growth Factor Receptor 

Epidermal growth factor receptor (EFGR) is a transmembrane glycoprotein belonging to the ErbB family of receptor tyrosine kinases (RTKs), which consist of ErbB-1 (EGFR), ErbB-2 (HER2/neu), ErbB-3 (HER3), as well as ErbB-4 (HER4) [51,52]. EGFR signaling is commonly altered in numerous human cancers due to *EGFR* gene amplification and/or overexpressions of protein, mutations, or in-frame deletions [53]. The regulation of EFGR action is critical for cancer prevention. The interaction between formononetin and EGFR WT/activating mutants ex vivo was examined via an in vitro pulldown assay. The findings show that both WT and mutant EGFRs can indeed attach to formononetin because EGFRs can be taken down by formononetin-conjugated Sepharose 4B beads, but not by Sepharose 4B beads on their own. According to the results of the in vitro kinase experiment, both formononetin and Osimertinib significantly reduce the kinase activity of activating mutants. Furthermore, formononetin, but not osimertinib, reduced EGFR WT kinase activity [54].

## 3. Role of Formononetin in Different Type of Cancers

**Figure 3:**
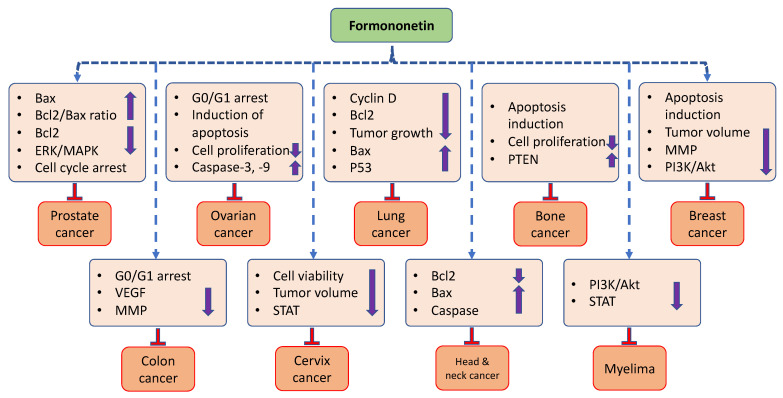
The anticancer potential of formononetin in different cancers.

The scientific literature shows that formononetin has an important role in cancer management through the modulation of various cell signaling molecules. Here, we summarized the potential role of formononetin in different types of cancers (Figure 3).

### 3.1. Prostate Cancer

The most frequently diagnosed form of cancer in men is prostate, and it is most prevalent in Western nations [55]. The proliferation of prostate cancer cells, such as LNCaP and the prostate cancer-3 (PC-3) cell line, were inhibited by formononetin at a high concentration (80 μM). However, this effect was found to be more marked in LNCaP as compared to PC-3 cells. Additionally, formononetin inhibited the extracellular signal-regulated kinase1/2 (ERK1/2) mitogen-activated protein kinase (MAPK) signaling pathway in a concentration-dependent way, which resulted in increased expression levels of Bcl-2-associated X (Bax) mRNA and protein, and induced apoptosis in LNCaP cells [17]. Formononetin played a major role in the inhibition of the growth of PC-3 (prostatic adenocarcinoma-3) cell in a concentration-dependent way, whereas this effect was not seen in human prostate epithelial cells (RWPE1). After the treatments, the apoptotic counts were effectively increased and formononetin treatment contributed to the decrease in Bcl-2 protein level and the increase in Bax expression in cancer cells, which improved the Bax/Bcl-2 ratio [14]. Formononetin was further found to reduce the viability of prostate cancer PC-3 cells and to induce apoptosis in a time- as well as concentration-dependent manner. Moreover, the expressions of Bcl-2 and long non-coding RNA (lncRNA) H19 (first discovered in lncRNA with a total length of 2.3 kb) [56] were markedly decreased as compared with the untreated group, whereas Bax was increased [57]. 

It was found that the percentage of cells that stayed in the G0/G1 phase was only 40% in the vehicle control group. However, formononetin-treated cells (20, 40, and 80 µM) demonstrated a gradual increase in the percentage of the G0/G1 phase at concentrations of 50%, 62% and 68%, indicating that formononetin exerts inhibitory effects on PC-3 cells’ growth via the induction of cell cycle arrest [8] (Table 2).

### 3.2. Breast Cancer

In 2020, breast cancer was one of the most-diagnosed cancers and the major cause of cancer-related deaths in women [57]. Chemotherapy and radiotherapy-based treatments of this cancer cause adverse effects. However, herbs or bioactive components of medicinal plants have played a significant role in breast cancer inhibition. In this scenario, formononetin has confirmed its role in breast cancer management. 

Taxol is an anticancer drug used for the treatment of different types of cancers, including breast cancer. MiR-199a-3p, a cancer-linked miRNA, is altered in many cancers. The mechanistic target of rapamycin (mTOR) is a dual-specificity protein kinase phosphorylating serine/threonine as well as tyrosine residues [58], and its role in cancer is known. It was noticed that MDA-MB-231/Taxol exhibited comparatively high drug resistance at a concentration of 0–7.5 μM/L, and Taxol slowly reduced drug resistance when the concentration was 10–15 μM/L. Furthermore, it was found that the concentration of formononetin showed low toxicity to MDA-MB-231/Taxol in the range of 5–20 μM/L, while formononetin at 40–80 μM/L showed significant cytotoxicity to the drug-resistant strain. In order to measure whether formononetin affects MDA-MB-231/Taxol cell growth through autophagy, autophagy-inhibitor 3-MA was used to treat the cells. It was observed that this autophagy inhibitor reduced MDA-MB-231/Taxol viability, and formononetin effectively suppressed MDA-MB-231/Taxol cell growth [59]. 

Mammalian target of rapamycin (mTOR) is a serine/threonine protein kinase in the PI3K-related kinase (PIKK) family and everolimus is an inhibitor of this pathway [60]. Everolimus acts more effectively against breast cancer cell growth in vitro as well as in vivo when combined with formononetin. Everolimus and formononetin together reduced tumor volume by twofold and decreased cell survival by 21.6%. This reveals that formononetin can synergistically enhance the tumoricidal effect of everolimus in human breast cancer cells. In cells treated with formononetin and everolimus, the apoptosis ratio increased by 27.9% [61]. With a concentration less than 160 µmol/L, formononetin had a negligible inhibitory effect on the viability of MDA-MB-231 and 4T1 cells (breast cancer cell lines). 4T1 and MDA-MB-231 cells’ migration and invasion were explored to be significantly suppressed when given nontoxic doses of formononetin. Formononetin also had a strong inhibitory effect on the phosphorylation of PI3K and Akt. Together, such results indicate that formononetin is involved in the inhibitory activity of breast cancer cell invasion and migration by reducing the expressions of MMP-2 and MMP-9 via the PI3K/AkT signaling pathway [35]. The proliferation of MDA-MB-231 and BT-549 cell lines was inhibited after treatment with formononetin in a dose-dependent manner, and this suppression peaked when formononetin concentrations of 40 and 80 μmol/L were used. Treatment with 40 µmol/L formononetin significantly decreased the migration and invasion of the MDA-MB-231 and BT-549 cell lines [62] (Table 2).

### 3.3. Cervix Cancer

Cervix cancer is the foremost cause of death, particularly in developing countries. In particular, te cervix cancer incidence is more common in Asia, Africa, and South America (regions in development), which might be due to insufficient resources, lack of screening programs, and access to healthcare [63,64]. Formononetin significantly and dose-dependently reduced the viability of HeLa cells. In addition, formononetin (50 µmom/L) treatment increased the rate of apoptotic cells to 42.11% and it increased the number of dead cells. The amount of ATP produced and overall oxygen saturation meaningfully decreased in comparison to the control group, as formononetin inhibited cancer cell viability. Nude BALB/c mice were used for the subcutaneous implantation of human cervical tumor cell HeLa for an in vivo study. It was reported that formononetin decreased tumor growth as compared to the control [65].

According to the present findings, formononetin inhibited the synthesis of the PD-L1 protein by reducing the expressions of STAT3 and MYC proteins. Formononetin also played a role in the inhibition of STAT3 activation through the JAK1/STAT3 pathway and significantly reduced the expression of the MYC protein via the RAS/ERK signaling pathway. Formononetin also promoted tumor cell apoptosis by suppressing PD-L1, and it prevented tumor cell growth, migration, and tube formation [44].

### 3.4. Ovarian Cancer

In ES2 and OV90 ovarian cancer cells, formononetin accelerated apoptosis and reduced cell proliferation through sub-G0/G1-stage arrest. In addition, it caused both cancer cells to produce reactive oxygen species and lose the potential of the mitochondrial membrane. Formononetin and pharmacological inhibitors (LY294002 or U0126) used to target the PI3k/Akt pathway were jointly administrated, and this resulted in additional anti-proliferative effects on both cancer cell types. The outcomes strongly suggest that formononetin can play an important role as an anticancer agent in cases of human ovarian cancer [66]. Formononetin treatment significantly reduced cell viability in ovarian cancer cells. In addition, formononetin suppressed cell proliferation via the induction of apoptosis. After receiving formononetin, it was found that ovarian cancer cells expressed apoptosis-related proteins, and that the ratio of Bax/Bcl-2 and the expression of caspase-3/9 proteins increased in a concentration-dependent way. Formononetin reduced the expression of MMP-2/-9 proteins as well as the phosphorylation level of ERK [67] (Table 2).

### 3.5. Osteosarcoma

Osteosarcoma is one of utmost common primary bone cancers that occurs in children and adolescents; the incidence of this cancer is low and occurs in about three per million people [68,69]. Despite the development in treatment approaches, the survival rate of this cancer is still low. Therefore, effective and safe modes of treatment are needed to manage this cancer. To ascertain the inhibitory effects of formononetin on the proliferation of human osteosarcoma cells, U2SO cells were exposed to formononetin at concentrations ranging from 0, 20, 40, and 80 µM for 0, 24, 48, and 72 h, and then evaluated by MTT assay. For the same concentration of formononetin, at longer time periods, the formononetin meaningfully inhibited the growth of cancer cells as compared to the control group. Additionally, after U2SO, cells were exposed to formononetin at a concentration of 80 µM; an apparent increase in the quantity of apoptotic cells was observed [15]. 

Formononetin encouraged PTEN expression, reduced the level of miR-214-3p, reduced cell viability, and started the apoptosis process. It has been reported that PTEN acts as a target of miR-214-3p, and formononetin increased the PTEN level by restricting miR-214-3p. A further investigation revealed that overexpressing miR-214-3p increased cell viability and reduced apoptosis in MG-63 cells by suppressing the expression of PTEN [40]. Additionally, the treatment of formononetin resulted in the significant inactivation of Akt and ERK, followed by the downregulation of Bcl-2, upregulation of Bax, and increased expression of caspase-3 [16] (Table 2).

### 3.6. Urinary Bladder Cancer

Formononetin effectively inhibited the proliferation of bladder cancer T24 cells in a concentration- and time-dependent manner. When exposed to formononetin, bladder cancer (T24) cells displayed apoptotic morphological variations and minimal invasiveness. Additionally, in formononetin-treated T24 cells, miR-21 expression was meaningfully reduced, followed by the enhancement of PTEN and p-Akt downregulation. Together, these outcomes suggest that formononetin shows an anticarcinogenic effect on bladder cancer based on in vitro investigations, which can be caused by the miR-21-initiated directive of the PTEN/Akt cascade [39].

### 3.7. Head and Neck Cancer

Formononetin significantly increased FaDu cell death. Additionally, formononetin-treated FaDu cells showed an increase in the characteristics of apoptosis, such as DNA fragmentation, chromatin condensation, morphological changes, and the size of the cell population going through apoptosis. Moreover, formononetin induced FaDu cell death by activating the caspase cascade, which involved both the mitochondria-dependent intrinsic and extrinsic apoptotic mechanisms that were facilitated by death receptors. In FaDu cells, the reduction in mitogen-activated protein kinases, such as extracellular signal-regulated kinase 1/2, nuclear factor-kB phosphorylation, and p38, could facilitate formononetin chemotherapeutic effects. In a FaDu cell xenograft animal model, oral formononetin administration inhibited tumor growth by increasing the expression of cleaved caspase-3 [70]. 

Formononetin played a role in the inhibition of the proliferation of both nasopharyngeal carcinoma (NPC) cells lines (CNE1 and CNE2), and it also caused CNE1 cells to undergo apoptosis in vitro. Treatment with formononetin significantly decreased the phosphorylation of Akt in CNE1 cells, whilst also increasing the phosphorylation of c-Jun N-terminal kinase/stress-activated protein kinase (JNK/SAPK) and p38MAPK [34]. Low-level formononetin stimulation of CNE2 cell proliferation was accompanied by a corresponding up and downregulation of Bax and Bcl-2 mRNA levels. The levels of the Bcl-2 and p-ERK1/2 proteins was shown to be increased. In addition, formononetin stimulated the proliferation of the CNE2 cell and inhibited the apoptosis of CNE2 cells, which was regulated by activating ERK1/2 signaling cascades [71] (Table 2).

### 3.8. Lung Cancer

The treatment with formononetin significantly inhibited the proliferation of two non-small cell lung cancer (NSCLC) cell lines, such as NCI-H23 and A549, in a time- and dose-dependent way. Moreover, it was established that formononetin treatment promoted cell apoptosis and induced G1-phase cell cycle arrest in NSCLC cells. On a molecular level, it was observed that formononetin exposure altered the levels of expression of the proteins p21, cyclin A, and cyclin D1 that are linked to cell cycle arrest. As a result of formononetin treatment, the apoptosis-linked cleaved proteins caspase-3, bcl-2, and bax became altered. Together, these findings establish that formononetin might be a possible chemo-preventive drug for lung cancer therapy via the induction of apoptosis and cell cycle arrest [10]. 

In vivo, ex vivo, as well as in vitro, formononetin inhibits the activity of WT as well as mutant EGFR (epidermal growth factor receptor) kinases. According to molecular modeling information, the ATP-binding pocket that is present in both mutant as well as WT epidermal growth factor receptors are where formononetin docks. Formononetin inhibits EGFR-Akt signaling, which in turn triggers GSK3β as well as induces Mcl-1 phosphorylation in non-small cell lung cancer cells. In a xenograft mouse model, formononetin inhibits the growth of tumors [54] (Table 2).

### 3.9. Brain Cancer

The glioma cell lines, including U87MG, T98G, and U251MG, were incubated with a series of formononetin concentrations ranging from 0 to 200 μM, and the results of the CCK-8 assay show that formononetin shows much less cytotoxicity in cancer cells between 0 and 100 µM. However, formononetin at concentrations of 150 and 200 µM significantly inhibited cell viability. As a result, 100 µM of formononetin was administered alongside doxorubicin. As a result, doxorubicin sensitivity was enhanced in glioma cells lines after co-administration with formononetin [72]. 

### 3.10. Myeloma

In multiple myeloma cells, formononetin increased the induction of apoptosis by bortezomib and it reduced proliferation. These effects were regulated by the activation of protein-1 (AP-1), phosphatidylinositol 3-kinase (PI3K)/AkT, and nuclear factor-B (NF-κB). The data showed that treatment with formononetin decreased the levels of numerous tumorigenic proteins involved in the progression and survival of myelomas. However, formononetin was found to block PI3K/Akt, NF-kB, and AP-1 activation in myeloma cells that were persistent [73]. 

Formononetin played a role in the inhibition of cell viability and induced apoptosis. Interestingly, formononetin was found to inhibit the constitutive activation of the signal transducer and transcriptional activator 3 (STAT3) (tyrosine residues 705 and 727) and STAT5 (tyrosine residues 694 and 699) in multiple myeloma cells, which was associated with the suppression of upstream kinases. Cell cycle arrest caused by formononetin was associated with the downregulation of STAT3-regulated anti-apoptotic, angiogenetic, and proliferative gene products, as well as caspase-3 activation and PARP cleavage. Without manifesting any significant negative effects, formononetin was administered intraperitoneally to reduce tumor growth in a multiple myeloma xenograft mouse model [45] (Table 2).

### 3.11. Colon Cancer

Formononetin-regulated angiogenesis and tumor cell invasiveness in colon cancer cells and tumor xenografts were examined. The results show that matrix metalloproteinases and vascular endothelial growth factor expression were reduced by formononetin. Additionally, it was observed that drug therapy improved the invasiveness of metastatic colon cancer cells. Moreover, in the tumor tissues obtained from the group receiving formononetin treatment, the number of proliferating cells and tumor size were noticed to be decreased. The serum vascular endothelial growth factor level was decreased in the drugged animals in comparison to the control groups [19]. Formononetin significantly reduced the ability of colon cancer cell lines SW1116 and HCT116 to proliferate and invade. According to the mechanistic studies, formononetin is able to inhibit the growth of colon cancer cells by downregulating the expression of the cell cycle-associated protein (cyclin D1) and causing cell cycle arrest at the G0/G1 checkpoint. Formononetin treatment further inhibited the expression of matrix metalloproteinases-2 and -9. Additionally, the inhibitory effects of formononetin were related with the signal transducer and activator of transcription 3 (STAT3) and phosphatidylinositol 3-kinase (PI3K)/protein kinase B (AkT) and signaling pathways [11]. 

Formononetin-treated human colorectal cancer cells (RKO cell line) were examined for the changes in endogenous expressions of gene/protein regulators. Compared to the control, formononetin inhibited RKO cell proliferation and played a role in the induction of apoptosis. In the meantime, formononetin lowered Bcl-2 protein expression and ERK1/2 phosphorylated level and upregulated Bax mRNA expression. Formononetin inhibited solid tumor growth in nude mice via reducing neoplastic NF-κB and TNF-α production [74] (Table 2).

### 3.12. Gastric Cancer

Formononetin has been shown to play a significant concentration-dependent role in the inhibition of gastric cancer cell proliferation. Formononetin was demonstrated to significantly reduce the ability of SGC-7901 and MGC-803 cells to form colonies. The migration and invasiveness of MGC-803 and SGC-7901 cells were further assessed to determine how formononetin affected the aggressive characteristics of gastric cancer cells. Evidently, formononetin (30, 50, and 80 μM) suppressed the ability of SGC-7901 and MGC-803 cells to migrate. In vitro, formononetin was found to control gastric cancer cell invasion in a concentration-dependent manner [75] (Table 2). Formononetin, a new derivative named as compound 10, was designed and synthesized using a molecular hybridization technique. According to the results, this derivative exhibit potent antiproliferative effects on SGC7901 cells. Through the Wnt/-Catenin and AkT/mTOR pathways, this derivative was shown to suppress the growth and migration of gastric cancer SGC7901 cells. According to the results of the in vivo tests, this derivative was found to effectively inhibit SGC7901 xenograft tumor growth without significantly reducing body weight [76].

**Table 2:** Role of formononetin in various types of cancer.

Cancer	Findings of the Study	Refs.
Prostate cancer	It inhibits the proliferation of LNCaP and PC-3 cells, whereas the most prominent effect was seen in LNCaP cells.	[17]
	Formononetin treatment contributed to increased Bax and reduced Bcl-2 protein level expressions in PC-3 cells, in that way resulting in the increasing Bcl-2/Bax ratio.	[14]
	It meaningfully inhibited the viability of PC-3 cells and indorsed apoptosis, and expressions of lncRNA, H19, and Bcl-2 were downregulated.	[57]
	Formononetin showed inhibitory activity against cancer cells in vivo and in vitro, which is connected to G1 cell cycle arrest by the inactivation of Akt/cyclin D1/CDK4.	[8]
Breast cancer	The combination therapy of formononetin and Taxol was found to be more effective in inhibiting drug resistance and autophagy.	[59]
	This compound increased the efficacy of everolimus in suppressing breast cancer cell growth. The combination of formononetin and everolimus decreased tumor volume and cell survival.	[61]
	Migration and invasion of MDA-MB-231 and 4T1 cells were suppressed. Furthermore, formononetin reduced the expressions of MMP-2, MMP-9, and increased the expression of the tissue inhibitor of metalloproteinase-1 (TIMP-1) and TIMP-2.	[35]
	It meaningfully decreased expressions of lncRNA AFAP1-AS1, CDK4, and Raf-1, whereas it increased miR-195 and miR-545 expressions in TNBC cells.	[62]
Cervix cancer	It played a role in the inhibition of the phosphorylation of AKT as well as presented apoptosis in a dose-dependent way. Additionally, formononetin decreased xenograft tumor growth in nude mice.	[65]
	This compound increased the activity of cytotoxic T lymphocytes as well as re-established their capability to kill tumor cells in a co-culture system of T cells as well as tumor cells.	[44]
Ovarian cancer	It reduced cell proliferation via sub G0/G1-phase capture and encouraged the loss of mitochondrial membrane potential as well as the generation of reactive oxygen species.	[66]
	It suppressed cell proliferation via the induction of apoptosis and reduced expression of MMP-2/9 proteins, as well as the phosphorylation level of ERK.	[67]
Bone cancer	This compound meaningfully inhibited the growth of cancer cells and caused an increase in the number of apoptotic cells.	[15]
	It reduced cell viability and caused apoptosis via regulating miR-214-3p. FN acted as a new treatment for MG-63 cells through enhancing the PTEN level via preventing the increase in miR-214-3p level.	[40]
	This compound activated the apoptosis of cancer cells, and treatment with this compound led to the inactivation of ERK as well as Akt.	[16]
Urinary bladder cancer	It meaningfully prevented the proliferation of bladder cancer in a time- and dose-dependent fashion.	[39]
Head and neck cancer	This compound initiated cancer cell death and involved death receptor-facilitated extrinsic as well as mitochondria-dependent intrinsic apoptotic pathways.	[70]
Lung cancer	The treatment promoted cell apoptosis and induced G1-phase cell cycle arrest.	[10]
	It suppressed WT and mutant epidermal growth factor receptor (EGFR) kinase activity. Treatment with formononetin enhanced the interaction between Mcl-1 as well as SCF^Fbw7^, which ultimately encouraged Mcl-1 ubiquitination and degradation.	[54]
Brain cancer	Combination treatment with formononetin reversed the doxorubicin-induced epithelial–mesenchymal transition in tumor cells.	[72]
Myeloma	The treatment reduced the levels of diverse tumorigenic proteins participating in myeloma progression. Remarkably, it was noted that formononetin blocked persistent PI3K/AKT, NF-κB, and AP-1 activations.	[73]
	It persuaded cell cycle arrest, decreased the expression of STAT3-regulated anti-apoptotic as well as angiogenetic gene products.	[45]
Colon cancer	Proliferation and invasion of colon carcinoma cell lines was significantly inhibited.	[11]
	It inhibited cancer cell proliferation and played a role in the induction of apoptosis. Moreover, formononetin lowered Bcl-2 protein expression and ERK1/2 phosphorylated level and upregulated Bax mRNA expression.	[74]
Gastric cancer	This compound evidently inhibited the migratory capabilities of cancer cells, and this compound dose-dependently controlled the invasion of gastric cancer cells in vitro.	[75]

## 4. Synergistic Effects of Formononetin with Anticancer Drugs

**Figure 4:**
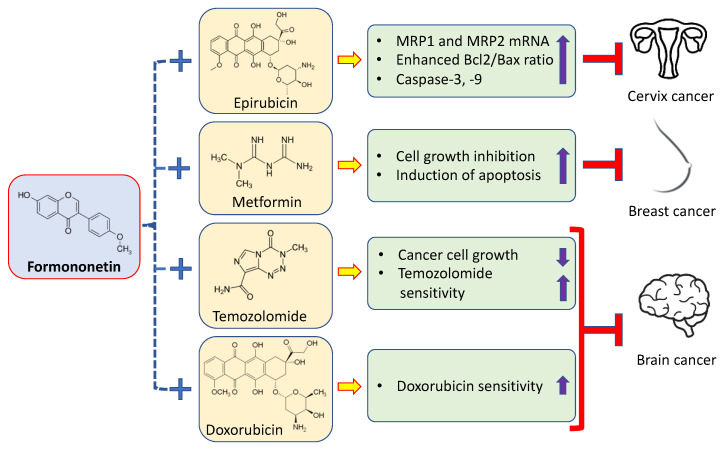
Synergistic effects of formononetin with anticancer drugs.

The commonly used cancer drugs cause adverse effects; drug resistance and such limitations restrict the benefits of these drugs in cancer treatment. However, anticancer drugs in combination with natural products or their bioactive ingredients show greater efficacy, low toxicity, and decrease drug resistance. The synergistic effects of formononetin on anticancer drugs are described accordingly (Figure 4). In ES2 and OV90 ovarian cancer cells, formononetin has been reported to increase apoptosis as well as reduce cell proliferation through sub-G0/G1-stage arrest. In cancer cells, the reduced phosphorylation of P90RSK, ERK1/2, P70S6K, S6, and AkT proteins, and enhanced phosphorylation of the P38 protein were found to be implicated in the formononetin-initiated regulation of both cell proliferation and apoptosis. When formononetin and pharmacological inhibitors (U0126 or LY294002) were administered together, more anti-proliferative consequences were seen in both types of human ovarian cancer cells [66]. 

Formononetin makes human cervical cancer HeLa cells more sensitive to the anticancer drug epirubicin. According to the findings, formononetin significantly increases the cytotoxic effects of epirubicin. Moreover, ROS levels were increased when formononetin and epirubicin were co-incubated. In addition, MRP1 and MRP2 mRNA expressions were both decreased by formononetin alone, or in combination with other treatments. Additionally, this result indicates that formononetin addition inhibits efflux transporter-mediated epirubicin resistance at different levels. 

The uptake of epirubicin into HeLa cells was significantly increased by this substance. Chromatin condensation, nuclear DNA fragmentation, and an increase in the sub-G1 and G2/M phases were signs that formononetin and/or epirubicin were the agents that started the apoptosis process. The combined treatment induced the activation of the apoptotic pathway of mitochondria, indicated through the loss of mitochondrial membrane potential, enhanced the Bax-to-Bcl-2 expression ratio, and activated caspases-9 and -3 [77] (Table 3). We examined how formononetin and metformin interact to promote the proliferation/growth of MCF-7 cells. As compared to untreated cells, 40 as well as 80 μM of formononetin significantly increased apoptosis and inhibited the proliferation of MCF-7 cells. Furthermore, the expressions of Bcl-2 mRNA and Bcl-2 and p-ERK1/2 proteins were significantly decreased by formononetin at concentrations of 80 and 40 μM. When 150 μM of metformin was added, formononetin cytotoxic effects were noticed to be more pronounced. Together, the use of metformin and formononetin led to a greater inhibition of cell growth and induced apoptosis in MCF-7 cells that was facilitated by the ERK1/2 signaling cascade [78] (Table 3).

**Table 3:** Synergistic effects of formononetin with anticancer drugs.

Cancer	Formononetin + Anticancer Drugs/Compounds	Findings	Refs.
Cervix cancer	Formononetin + epirubicin	Formononetin significantly enhanced the cytotoxicity of epirubicin. Moreover, the co-incubation of epirubicin with formononetin increased ROS levels, including hydrogen peroxide and superoxide free radicals.	[77]
Breast cancer	Formononetin + metformin	The combination use of metformin and formononetin increased cell growth inhibition and induced apoptosis in MCF-7 cells mediated via the ERK1/2 signaling pathway.	[78]
Brain cancer	Formononetin + doxorubicin	Doxorubicin sensitivity was enhanced in glioma cells after co-administration with formononetin.	[72]
	Formononetin + Temozolomide	Formononetin or temozolomide alone inhibited the growth of C6 glioma cells in a dose-dependent way and formononetin in combination with temozolomide had a synergistic effect on C6 cells. Combination drugs decreased the expression.	[79]
	Formononetin and calycosin + temozolomide	Formononetin and calycosin (FMN/CAL) increased the inhibition of temozolomide on the growth as well as infiltration of C6 glioma.	[80]

CCK-8 assay was used to evaluate the cell viability treated with doxorubicin, either separately or with formononetin, to evaluate a synergistic cytotoxicity of doxorubicin in the presence of formononetin. The findings reveal that the doxorubicin sensitivity is enhanced in glioma cells (U251MG, U87MG, and T98G) after co-administration with formononetin. These findings suggest that formononetin might show glioma cells to be more sensitive to doxorubicin. Moreover, the administration of doxorubicin significantly enhanced the expression of vimentin, and E-cadherin decreased in U87MG cells. However, combined treatment with formononetin increased E-cadherin and reduced vimentin expression levels, showing that EMT induced by doxorubicin was reversed by formononetin in glioma cells [72]. Formononetin or temozolomide alone inhibited the growth of C6 glioma cells in a dose-dependent way, and formononetin in combination with temozolomide had a synergistic effect on C6 cells. Combination drugs decreased the expression of Bcl-2, enhanced the expression of Bax, cleaved caspases-3 and -9, and induced apoptosis tumor cells [79] (Table 3). 

Formononetin and calycosin (FMN/CAL) increased the inhibitory potential of temozolomide on C6 glioma growth and infiltration. The metabolomic outcome showed that the temozolomide sensitization of formononetin and calycosin (FMN/CAL) mostly involved one metabolic pathway, two metabolites in tumor tissues, five metabolic pathways and four metabolites in cells, and seven metabolic pathways and eight metabolites in serum. A further network pharmacological analysis showed that NOS2 was indeed a great, promising target for formononetin and calycosin (FMN/CAL) to regulate metabolism in temozolomide-treated C6 glioma cells. TNF-α secretion in the tumor region was decreased by FMN/CAL, and NOS2 expression in tumor tissues and cells were also decreased. This finding establishes a rationale for the use of FMN/CAL as a glioma-adjuvant treatment by demonstrating that they can increase malignant glioma sensitivity to temozolomide through suppressing NOS2-dependent cell survival [80] (Table 3).

## 5. Pharmacokinetics, Bioavailability, and Approaches to Improve Formononetin Delivery

The therapeutic effect of natural compounds or chief ingredients of medicinal plants have proven to be effective in health management [81,82,83,84,85,86], including cancer, through modulating cell signaling pathways, including angiogenesis, inflammation, apoptosis, and other cell signaling molecules [87,88,89]. Formononetin has confirmed its role in disease management. However, its role is limited due to its poor absorption and bioavailability, low solubility, rapid removal, and metabolism factors. Formononetin and its glycoside ononin were studied to determine their in vitro absorption, pharmacokinetics, and bioavailability. The data showed that formononetin had a higher systemic exposure than ononin following an oral administration to rats. Ononin and formononetin had oral bioavailability rates of 7.3% and 21.8%, respectively. Moreover, ononin as well as formononetin both exhibited superior absorption in small intestine segments than in large intestine segments. However, formononetin showed better intestinal permeability in all segments, in comparison to ononin [90]. 

Formononetin showed high permeability through an investigation called the parallel artificial membrane permeability assay (PAMPA) at pH 4.0 and 7.0. Moreover, the plasma protein binding of formononetin was found to be 93.61 ± 0.44% and 96.14 ± 0.15% at the evaluated doses of 50 and 150 ng/mL, respectively. The bioavailability of free/relatively unchanged formononetin was explored to be only about 3%, which was considered to be low. In addition to having a significant apparent volume of distribution (14.16 L/kg), formononetin was discovered to have a high clearance rate (5.13 L/h/kg). Furthermore, it was revealed that the scores of isoflavone glucuronides/sulfates were significantly higher than those of the respective aglycones [91]. 

Nano-formulations for drug delivery have been described and their therapeutic effects have been noticed to be better than native compounds. Previous results have shown that nano-formulation-based approaches can be used as a delivery approach to overcome the bioavailability issue of formononetin. Innovative formononetin–dithiocarbamate derivatives were drafted, produced, and evaluated for their ability to inhibit the proliferation of cancer cell lines (MGC-803, EC-109, and PC-3). This study, which looked at 14 structural class variants, found the first SAR (structure–activity relationship) for the formononetin–dithiocarbamate scaffold. In this group of analogs, the compound with the highest inhibitory activity in the case of PC-3 cells was tert-butyl 4-(((3-((3-(4-methoxyphenyl)-4-oxo-4H–chromenyl)oxy)propyl)thio)carbonothioyl)piperazine-1-carboxylate (8i)). The cellular mechanism result shows that 8i regulates the expression of G1 checkpoint-related proteins and arrests cell cycles at the G1 phase in a concentration-dependent fashion. Additionally, 8i might inhibit cell growth through the MAPK signaling pathway as well as the inhibition of migration through Wnt pathway in PC-3 cells [92]. Cytotoxicity was evaluated for formononetin nitrogen mustard derivatives. The results show that several of the innovative derivatives show stronger cytotoxicity than alkeran [93] (Table 4). Three hybrids of podophyllotoxin and formononetin were examined for anticancer efficacy. Its role in the inhibition of invasion and migration in A549 cancer cells was noticed [94]. Multiwalled carbon nanotube–formononetin (MWCNT-FMN) material was investigated for sustained release and inducing apoptosis in HeLa cells. The loading capacity was 12.05% ± 0.20% and entrapment efficacy was 28.77% ± 0.15%. The release of MWCNT-FMN was found to be sustained. Formononetin (FMN), MWCNT with a carboxylic group (MWCNT-COOH), and formononetin attached to MWCNT were tested for cytotoxicity in vitro (MWCNT-FMN). The results show that none of the materials under test conditions present any appreciable consequences regarding the viability and proliferation of mouse fibroblast 3T3 cells; however, the three samples’ abilities to inhibit cell growth in HeLa cells is concentration-dependent [95]. To increase the potentiality of the formononetin (FN) antitumor effect, a passive targeting FN-contained formulation was developed. FN-containing vitamin-E d-α-tocopheryl polyethylene glycol 1000 succinate (vitamin E TPGS or TPGS)/phospholipid micelles were produced. The outcomes show that the prepared formononetin micelles have higher stability, and the average diameter of each particle is reported to be 111.91 ± 5.82 nm. In comparison to free formononetin, formononetin micelles showed increased cellular efficiency for uptake and cytotoxicity. Furthermore, DiR micelles were deposited more rapidly at the tumor site as opposed to free or unbound DiR micelles. In tumor-bearing mice treated with formononetin micelles, the tumor inhibition rate and biosafety were significantly higher than that observed in mice treated with free formononetin [96] (Table 4).

For the evaluation of prostate cancer therapy, the efficacy of the synergistic combination of docetaxel (DTX) and formononetin (FMN) combined as a nanoformulation was analyzed. Hyaluronic acid (HA) and epidermal growth factor receptor-targeted peptide (GE11) dual ligands were applied to modify the nano-systems. HA/GE-DTX/FMN-NPs showed a cellular uptake efficiency of 59.6% and a more efficient inhibition effect on prostate cancer (PC3) cells compared with single-ligand-modified NPs and free drugs. These NPs showed greater tumor inhibition potential as compared to their single drug-loaded counterparts and free drugs [97]. 

A nanoscale amphiphilic and cartilage-targeting polymer-drug delivery system using formononetin (FMN)-poly(ethylene glycol) (PEG) (denoted as PCFMN) was synthesized, as prepared by the PEGylation of FMN, followed by coupling with a cartilage-targeting peptide (CollBP). The results demonstrate that PCFMN is almost spherical, with an average diameter of about 218 nm. The in vitro test using IL-1β-stimulated chondrocytes showed that PCFMN was biocompatible and enhanced anabolic genes, while at the same time, it decreased the catabolic genes of the articular cartilage. The therapeutic effects in vivo indicated that PCFMN could effectively reduce the progression of OA [98]. 

**Table 4:** Formononetin-based nano-formulations and their roles in cancer.

Derivatives/Nano Formulation	Outcome of the Study	Refs.
Formononetin–dithiocarbamate derivatives	Derivatives might inhibit cell growth through MAPK signaling pathway as well as the inhibition of migration through the Wnt pathway in PC-3 cells	[92]
Formononetin nitrogen mustard derivatives	The results show that several of the innovative derivatives show greater cytotoxicity than alkeran	[93]
Multi-walled carbon nanotube–formononetin	Formononetin (FMN) and multiwalled carbon nanotube– formononetin (MWCNT-FMN) can induce apoptosis in HeLa cells; in the meantime, the cells display an effective reactive oxygen species signal	[95]
FN-containing vitamin-E d-α-tocopheryl polyethylene glycol 1000 succinate	Formononetin micelles improve the cellular uptake and enhance cell cytotoxicity compared to free formononetin	[96]
Formononetin-2-hydroxypropyl-β-cyclodextrin inclusion complex-loaded PLGA nanoparticles	The in vitro cytotoxicity shows that formononetin-2-HPβ-CD-NP displays anticancer activity in MCF-7 and Hela tumor cells compared to free formononetin	[99]
Hydroxypropyl-β-cyclodextrin (HP-β-CD) modified carboxylated single-walled carbon nanotubes	Antitumor activity of carboxylated single-walled carbon nanotube (CD-SWCNTs)–FMN is stronger than that of free formononetin	[100]

Formononetin-2-hydroxypropyl-β-cyclodextrin inclusion complex-loaded PLGA nanoparticles (FMN-2-HPβ-CD-NP) were prepared. The in vitro release showed that FMN-2-HPβ-CD-NP presents a sustained release effect. The in vitro cytotoxicity showed that formononetin-2-HPβ-CD-NP exhibited anticancer activity in MCF-7 and Hela tumor cells compared to free formononetin [99] (Table 4). 

To increase the biocompatibility and reduce the toxicity of carbon nanotubes as a formononetin delivery system, a composition of carboxylated single-walled carbon nanotubes (CD-SWCNTs) was prepared using hydroxypropyl-cyclodextrin (HP-CD). According to an in vitro cytotoxicity assay, carboxylated single-walled carbon nanotubes (CD-SWCNTs)–FMN have stronger antitumor activities than free formononetin [100] (Table 4).

## 6. Safety and Toxicity of Formononetin

The toxicity of sodium formononetin-3′-sulphonate (Sul-F), a water-soluble derivate of formononetin, was examined in dogs after 90-day intravenous infusion. Dogs were treated with this derivates at different dose, such as 0, 33.3, 100, and 300 mg/kg. No mortality, ophthalmic abnormalities, or treatment-associated findings in body weight, hematology, clinical chemistry, and histopathological examinations were noticed. However, a white crystal (non-metabolic Sul-F), transient vomiting, as well as recoverable vascular stimulation were noticed in 300 mg/kg/day Sul-F treated dogs. Under the circumstances, the no-observed-adverse-effect level for Sul-F was 100 mg/kg in dogs [101]. Acute toxicity level of sodium formononetin-3′-sulphonate (Sul-F) after intravenous administration in rats and dogs was evaluated. Animals were intravenously given Sul-F at the maximum dosages, individually, of 2000 and 1000 mg/kg in rats and dogs, respectively. The results reveal that no Sul-F related clinical signs of toxicity or mortality were seen in rats. Furthermore, a white crystal and non-metabolic Sul-F were noticed after urine volatilization in Sul-F-treated animals (rats and dogs). However, neither biochemical findings nor histopathological changes due to Sul-F treatment were noticed in tests [102].

We investigated whether formononetin, a phyto-oestrogen, and its metabolites, daidzein and equol, affected the production of interleukin-4 (IL-4), a pro-inflammatory cytokine link with allergic immune responses in primary CD4^+^ T cells and EL4 T lymphoma cells. Formononetin, daidzein, and equol meaningfully increased IL-4 production from both CD4^+^ T and EL4 cells in a dose-dependent way. These results advocate that phyto-oestrogens and some of their metabolites may enhance allergic responses through the enhancement of IL-4 production in T cells [103].

## 7. Clinical Trials of Formononetin

In vivo and in vitro studies confirmed that formononetin plays a significant role in cancer prevention through the modulation of various cell signaling pathways. researchers are exploring the clinical uses of flavonoids as anticancer agents in different clinical trials, at present. A short report based on the clinical trials of this compound is available. The effect on bone density of a red clover-derived isoflavone supplement that provided a daily dose of biochanin A (26 mg) A, formononetin (16 mg), genistein (1 mg), and daidzein (0.5 mg) for 1 year was examined. The effects of these compounds on the biochemical markers of bone turnover as well as body composition were also evaluated. Loss of lumbar spine bone mineral content and bone mineral density were meaningfully reduced in the women eating the isoflavone supplement than in those taking the placebo. There were no substantial treatment effects on hip bone mineral content or bone mineral density, markers of bone resorption, or body composition, while bone formation markers were meaningfully increased in the intervention group compared with the placebo in postmenopausal women [104]. Plasma lipids were not meaningfully affected. 

A significant cardiovascular risk factor, arterial compliance, which reduces with menopause, was meaningfully improved with red clover isoflavones containing genistein, daidzein, formononetin, and biochanin [105]. Thirty-eight patients were enrolled after the diagnosis of prostate cancer. Before surgery, 20 men consumed 160 mg/day of red clover-derived dietary isoflavones, comprising a mixture of formononetin, genistein, daidzein, and biochanin A. Serum PSA, biochemical factors, and testosterone were examined, and clinical and pathological parameters were noticed. There were no substantial differences between pre- and post-treatment serum PSA, serum testosterone, or biochemical factors in the treated patients. Apoptosis in radical prostatectomy specimens from treated patients was meaningfully higher than in control subjects, specifically in regions of low- to moderate-grade cancer. No adverse events related to the treatment were reported. This report suggests that dietary isoflavones may halt the progression of prostate cancer via encouraging apoptosis in low- to moderate-grade tumors [106].

## 8. Conclusions

The dysregulation of several important signaling cascades, including inflammation, JAK/STAT pathway, PI3K/Akt/mTOR pathway, MAPK pathway, tumor suppressor gene, Wnt/-catenin, and cell cycle and apoptotic pathways, were reported to be associated with the molecular basis of different cancers. Therefore, modulating signal transduction pathways can lead to the regulation of cell survival, proliferation, and metastasis of cancer cells. Formononetin is a type of isoflavone and its role in cancer management was discussed. the mechanism of action of formononetin in the management of different cancers was confirmed via the modulation of cell signaling molecules. Moreover, its anticancer potential was rooted in the modulation of inflammation, cell cycle, apoptosis, PI3K/Akt/mTOR pathway, angiogenesis, tumor-suppressor gene, and others cell signaling pathways. Therefore, comprehensive studies based on in vivo or clinical studies should be accomplished to reconnoiter the meticulous mechanism of action of formononetin in cancer prevention and treatment.

## Data Availability

Not applicable.
